# Revisiting the evidence for genotoxicity of acrylamide (AA), key to risk assessment of dietary AA exposure

**DOI:** 10.1007/s00204-020-02794-3

**Published:** 2020-06-03

**Authors:** Gerhard Eisenbrand

**Affiliations:** University of Kaiserslautern, Germany (Retired), Kühler Grund 48/1, 69126 Heidelberg, Germany

**Keywords:** Acrylamide, Glycidamide, Mode of action, Non genotoxic, Process related contaminants, Dietary exposure

## Abstract

The weight of evidence pro/contra classifying the process-related food contaminant (PRC) acrylamide (AA) as a genotoxic carcinogen is reviewed. Current dietary AA exposure estimates reflect margins of exposure (MOEs) < 500. Several arguments support the view that AA may not act as a genotoxic carcinogen, especially not at consumer-relevant exposure levels: Biotransformation of AA into genotoxic glycidamide (GA) in primary rat hepatocytes is markedly slower than detoxifying coupling to glutathione (GS). Repeated feeding of rats with AA containing foods, bringing about uptake of 100 µg/kg/day of AA, resulted in dose x time-related buildup of AA-hemoglobin (Hb) adducts, whereas GA-Hb adducts remained within the background. Since hepatic oxidative biotransformation of AA into GA was proven by simultaneous urinary mercapturic acid monitoring it can be concluded that at this nutritional intake level any GA formed in the liver from AA is quantitatively coupled to GS to be excreted as mercapturic acid in urine. In an oral single dose–response study in rats, AA induced DNA *N*^7^-GA-Gua adducts dose-dependently in the high dose range (> 100 µg/kg b w). At variance, in the dose range below 100 µg/kg b.w. down to levels of average consumers exposure, DNA *N*^7^ -Gua lesions were found only sporadically, without dose dependence, and at levels close to the lower bound of similar human background DNA *N*^7^-Gua lesions. No DNA damage was detected by the comet assay within this low dose range. GA is a very weak mutagen, known to predominantly induce DNA *N*^7^-GA-Gua adducts, especially in the lower dose range. There is consensus that DNA *N*^7^-GA-Gua adducts exhibit rather low mutagenic potency. The low mutagenic potential of GA has further been evidenced by comparison to preactivated forms of other process-related contaminants, such as *N*-Nitroso compounds or polycyclic aromatic hydrocarbons, potent food borne mutagens/carcinogens. Toxicogenomic studies provide no evidence supporting a genotoxic mode of action (MOA), rather indicate effects on calcium signalling and cytoskeletal functions in rodent target organs. Rodent carcinogenicity studies show induction of strain- and species-specific neoplasms, with MOAs not considered likely predictive for human cancer risk. In summary, the overall evidence clearly argues for a nongenotoxic/nonmutagenic MOA underlying the neoplastic effects of AA in rodents. In consequence, a tolerable intake level (TDI) may be defined, guided by mechanistic elucidation of key adverse effects and supported by biomarker-based dosimetry in experimental systems and humans.

## Introduction

Acrylamide (AA) is one of several so-called process-related contaminants (PRCs) occurring in heat-processed food worldwide. Further dietary PRCs of similar widespread occurrence encompasses glycidol and glycidol esters, chlorinated propanols (MCPD) and their esters, furans and substituted furans and, depending on the type of process and the temperatures applied to food, heterocyclic aromatic amines (HAA), *N*-Nitroso compounds (NOC) and polycyclic aromatic hydrocarbons (PAH). This list may not be exhaustive and further PRC may come into focus.

Of note, assembling these contaminants into one group is merely reflecting their mode of generation. They are formed from food constituents during the various heat treatment processes foods may be exposed to. These include cooking, microwaving, frying, baking, grilling, roasting, smoking, and/or other forms of industrial and household processing under heat.

In all other aspects, especially with regard to structures, exposure levels, mechanisms of biological action, and potency there are substantial differences within PRCs. It appears appropriate to differentiate a group of contaminants of high genotoxic, mutagenic and/or carcinogenic potency, such as the HAA, PAH, and NOC from the other compounds of comparatively lesser biological potency.

Historically, certain high potency genotoxic compounds have been detected in food and human–environment many years before the more recently discovered PRCs. As a consequence, mitigation measures have been developed and installed much earlier. It is therefore fair to state that as a consequence of more than 50 years of sustained mitigation and consumer education, dietary exposure to high potency PRCs in most cases decreased down to levels bringing about margins of exposure (MOE) close to or exceeding 10 000. The MOE approach normally utilizes a reference dose from an animal study associated with a low but measurable reponse which is compared to dietary intake estimates in humans (EFSA 2005). A MOE of 10 000 is equivalent to exposure levels not considered by the European Food Safety Authority (EFSA) to imply a relevant health risk to consumers. In contrast, mitigation measures and consumer information for some of the lower potency PRCs have been developed and implemented essentially only within the last two decades.

In 2002, AA contamination was discovered in a range of heat-processed foods. AA was found to arise during cooking, frying, roasting, baking, and to originate from natural precursors, known as innocuous food constituents. The main reaction occurs by heat-induced Maillard reaction of reducing carbohydrates with asparagine (Tareke et al. 2002). Food contamination data monitored during subsequent years reflect research on mitigation measures and their continuous implementation. Mitigation endeavors and communication to the consumer by health authorities and industry essentially followed directions agreed upon in so-called mitigation toolboxes, e.g. the Acrylamide Toolbox (Food Drink Europe 2019). This has been met with some success, especially with respect to the reduction of peak contaminations. Yet, average dietary consumer exposure in Europe has been estimated to correspond to MOE values within a range of about < 100 to < 500. The MOE was defined in the case of AA as the BMDL_10_ of 0.17 mg/kg/day divided by the consumer’s of 0.4–3.4 μg/kg bw/day. The BMDL_10_ was taken as the point of departure (POD) from a mouse carcinogenicity study, corresponding to the modelled dose rate associated with the lower bound confidence interval of a 10% enhanced Harderian gland tumor response (EFSA 2011, 2015).

Intriguingly, such a MOE range is orders of magnitude lower than the target MOE of 10 000 for genotoxic carcinogens considered of low public health relevance. Hence the continuing endeavor to reduce consumers’ exposure further, along with the ALARA principle (as low as reasonably achievable), cast into legal regulation by the European Union concerning mitigation of AA contamination in food (EU 2017).

During recent years toxicological risk assessment has experienced literally quantum jumps in methodology and predictive power. The advent of extremely powerful, ultrasensitive, and dependable analytical techniques, in conjunction with modern methods of cell and tissue biology, stem cell technology, and advanced in silico evaluation methods together have driven this spectacular progress. It encompassed, amongst others, continuous refinements in development and application of quantitative structure–activity relationships (QSAR), quantitative in vitro-in vivo extrapolation (QVIVE), physiologically based pharmacokinetic (PBBK) modelling, as well as combined omics technologies. Moreover, novel findings were provided from biomarker monitoring under experimental settings or in well-controlled human intervention studies. Altogether this has contributed to bring about deepened insights with respect to the elucidation of adverse outcome pathways and MOAs of agents like AA, considered genotoxic at consumer-relevant exposure levels.

### Hazard identification and characterization of AA

#### Metabolism and supposed mechanism of action

AA is well absorbed and rapidly distributed systemically. In addition to directly reacting with nucleophilic groups of plasma proteins and cellular constituents, it is extensively metabolized, primarily in the liver and predominantly by coupling to glutathione (GS), either spontaneously and/or mediated by glutathione-S-transferases. As a Michael reactant, AA avidly adds to nucleophilic centers, such as mercapto- or amino-groups of structural and soluble plasma proteins, including the N-terminal valine of hemoglobin (Hb). The generated Hb adducts can serve as biomarkers reflecting long term exposure since they build up during the about 3–4 month lifetime of animal/human erythrocytes. By contrast, GS adducts are metabolically trimmed into *N*-acetyl cysteine thioethers, known as mercapturic acids (MA) which are almost completely excreted in the urine within about 48 h after exposure. They, therefore, are preferentially utilized as short term exposure biomarkers and do not accumulate.

**Fig. 1:**
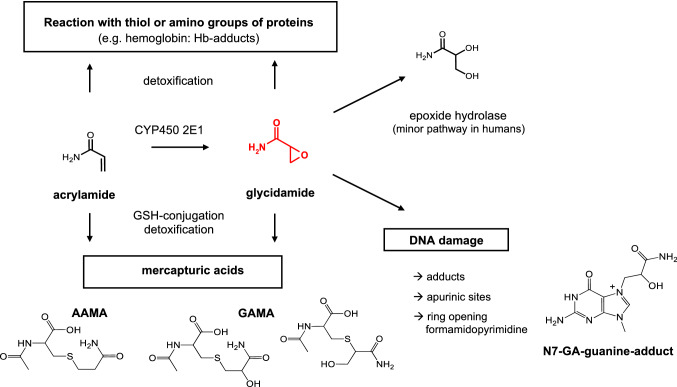
Major metabolic pathways of AA in the rat (reprinted from Hartwig et al. 2020)

A second major AA biotransformation pathway consists of cytochrome P450 2E1 (CYP2E1)-mediated epoxidation into 2,3-epoxypropanamide, known as glycidamide (GA). GA is considered to be genotoxic since it can damage the DNA by covalent binding to nucleophilic centers, primarily to the nitrogen in position 7 of the DNA base guanine (*N*^7^-Gua). Metabolic formation of GA is considered as activating biotransformation responsible for genotoxicity, mutagenicity, and carcinogenicity of the parent molecule, AA. The *N*^7^-Gua adduct, *N*^7^-(2 carbamoyl-2-hydroxy ethyl) guanine (*N*^7^-GA-Gua) is by far the most abundant DNA adduct derived from GA by covalent interaction. In contrast to GA adducts, covalent DNA adducts of AA itself have never been detected in vivo or in vitro in animal or human tissues. Figure 1 summarizes the relevant biotransformation pathways AA is undergoing in mammalian systems.

Of note, humans have been found to be less proficient than rodents in activating AA metabolically to the genotoxic metabolite GA, whereas detoxifying biotransformation, especially coupling of AA and GA to GS is more efficient in humans than rodents (Berger et al. 2011; Fennell and Friedman 2005; Fuhr et al. 2006). This species-related differential biotransformation, favoring detoxification in humans as compared to rodents, adds to the findings described below, showing that already in rat primary hepatocytes detoxification is faster than toxification at close to physiological conditions.

In an in vitro study, hepatic biotransformation was studied in primary rat hepatocytes incubated with AA in a wide range of concentrations (0.2–2 000 µM). Formation of GA from AA was measured in the medium, as well as the generation of the GS adduct, AA-GS. In addition, the formation of *N*^7^-GA-Gua was also searched for. The formation of AA-GS was linear with AA concentration and incubation time and became detectable already at 0.2 µM (4 h incubation). In contrast to AA-GS, GA was not detected before 16 h incubation at 10-fold higher AA concentration (2 µM). In summary, the rate of AA-GS formation was found to be about 1.5–3 times faster than that of CYP-mediated GA formation. DNA *N*^7^-GA-Gua adducts were found only at the highest AA concentration tested and after extended incubation times (2000 µM, 24 h), conditions far from being relevant to those expected to result from consumer’s exposure. Altogether, at more physiological conditions, especially at low AA concentrations and incubation times better-reflecting conditions related to consumer exposure, evidence was compelling that in the rat liver detoxifying formation of GS adducts is up to about 3 times faster than GA formation (Watzek et al. 2013).

#### Non-neoplastic effects

Animal studies have been conducted in various species, including rodents, cats, dogs, and monkeys at a range of dosages and routes of repeated application. Key observations were peripheral neurotoxicity, adverse effects to the testes, atrophy of skeletal musculature, and further, rather unspecific toxicities observed at relatively high repeated dosage levels (≫ 1 mg/kg bw/day).

The EFSA CONTAM Panel identified four key outcomes of AA toxicity, i.e. neurotoxicity, effects on male reproduction, developmental toxicity, and carcinogenicity. Benchmark dose (BMD) analysis was performed using experimental data on neurotoxicity and carcinogenicity. For neurotoxicity, the lowest BMDL_10_ was 0.43 mg/kg bw/day, derived from the incidences of peripheral (sciatic) axonal nerve degeneration in male F344 rats exposed in drinking water for 2 years. Since this was lower than the no observed adverse effect levels (NOAELs) for adverse effects on male reproductive parameters and for developmental toxicity, the BMDL_10_ for neurotoxicity was selected as a conservative reference point and point of departure (POD) for risk assessment of non-neoplastic effects (EFSA 2015).

#### Neoplastic effects

In long term studies, AA has exerted neoplastic effects in multiple tissues of rodents, in a dose range > 0.5 mg/kg bw/day. These essentially included the enhanced formation of mammary gland adenomas and fibroadenomas, thyroid follicular cell adenomas/carcinomas, and mesotheliomas of testes (tunica vaginalis) in Fisher (F344) rats. In mice, neoplastic effects encompassed Harderian gland tumors, together with tumors of mammary gland, lung, ovary, skin, and stomach/forestomach papillomas/carcinomas (summarized in EFSA 2015).

The EFSA CONTAM panel has selected the lowest BMDL_10_ of 0.17 mg/kg bw/day, derived from data on the incidence of Harderian gland tumors in male B6C3F1 mice continuously exposed to AA for 2 years. Although a Harderian gland is absent in humans, the rationale for this selection was

1. the consideration that this tissue was most sensitive in rodent bioassays to detect effects of genotoxic carcinogens and
2. Taking into account that target tissues for tumor formation by a given genotoxic carcinogen may differ between rodents and humans

The EFSA CONTAM Panel considered the MOE approach appropriate for risk characterization of neoplastic effects of dietary exposure to AA and derived MOE values ranging from about < 100 to < 500.

Of note and at variance with the aforementioned rodent carcinogenicity studies in mice and F344 rats primarily evaluated by EFSA, a two year carcinogenicity study carried out in a different rat strain (Wistar Han rats) brought about markedly divergent results (Maronpot et al. 2015). The design of this high quality and guideline compliant study encompassed transplacental exposure of pregnant animals from gestation day 6 until delivery, followed by continuous longterm treatment of the offspring. AA was applied in drinking water at a dosage range of 0.5–3 mg/kg bw/day. Male and female F1 offspring animals remained with the dams until day 21 of lactation and were randomized after weaning to the respective treatment subsets within each group. Offspring animals were continuously AA exposed at the same dosage until postnatal day 722.

A dose-related increase in thyroid follicular cell neoplasms in males and females was observed. Also, mammary fibroadenomas were found enhanced in the two highest dosage groups. These were observed in the majority of cases in animals surviving to the 24 month terminal dissection (Maronpot et al. 2015). However, no testes tunica vaginalis mesothelioma (TVM) and only one Leydig cell tumor (LCT) were observed, indicating a clear strain difference between F344 and Wistar Han rats (Maronpot et al. 2015).

The development of TVM is considered to reflect a specific susceptibility of the F344 rat to Leydig cell tumors with secondary induction of TVM. Given their high spontaneous background incidence and species-specific biology TVM responses in F344 rat carcinogenicity studies, -along with LCT and mononuclear cell leukemia have been rated inappropriate tumor types for human health risk assessment, lacking relevance in predicting human carcinogenicity (Maronpot et al. 2009, 2015, 2016; Shipp et al. 2006). Other possible treatment-related neoplasms identified in previous F344 rat carcinogenicity studies did not occur in Wistar Han rats, including those of the clitoral gland, cardiac schwannomas, islet cell carcinomas, or oral cavity tumors.

Rat mammary gland fibroadenomas are typically not considered precursors of malignant mammary adenocarcinomas. In the study of Maronpot et al. (2015), the overall incidence of mammary gland fibroadenomas in females was not statistically significant and within the published control range for Wistar rats (Maronpot et al. 2015). For mammary fibroadenomas in rats the luteotrophic effect of age-associated prolactinaemia is supposed to be causative. This mode of action is considered not likely relevant to women where prolactin is not luteotrophic (Ben-Jonathan et al. 2008; Maronpot et al. 2009, 2015; Neumann 1991). Likewise, in a review on spontaneous neoplasms in control Wistar rats covering 10 years of observation, amongst other neoplasms a predilection to pituitary neoplasms and mammary fibroadenomas in females was noted (Poteracki and Walsh 1989).

Thyroid follicular cell neoplasms occurred late in this study (Maronpot et al. 2015). Follicular cell neoplastic responses had also been documented in previously conducted AA carcinogenicity studies in Fisher 344 rats (Beland et al. 2013; Friedman et al. 1995; Johnson et al. 1986). Thyroid follicular changes are known to be associated with rat-specific thyroid hormone homeostasis which differs markedly from thyroid follicular cell hormonal physiology in man (Alison et al. 1994; Capen 1997). At variance to humans, rats are uniquely susceptible to developing thyroid follicular cell neoplasms. This is known to reflect marked differences in plasma thyroid hormone turnover, preferentially resulting from a lack of thyroid-binding globulin in rodents. As a consequence, rodent thyroid is less proficient than the human thyroid to maintain thyroid hormone homeostasis. Moreover, the halflife of thyroid hormones T4 and T3 is very much shorter (about tenfold) in rodents than in humans and thyroid-stimulating hormone (TSH) levels are much higher in rodents. Thus, rodent thyroid is on a markedly higher activity level than its human counterpart which is supposed to make rats considerably more susceptible to neoplastic effects than humans (Alison et al. 1994; Dourson et al. 2008; Maronpot et al. 2015).

A role for hormonal dysregulation affecting the pituitary–thyroid axis and the rat specific thyroid hormonal milieu is generally accepted as an explanation for follicular cell neoplasia in rat carcinogenicity studies and is considered a rat-specific response (Alison et al. 1994; Bartsch et al. 2018; Capen 1997; Capen and Martin 1989; Khan et al. 1999; Maronpot et al. 2009, 2015; Neumann 1991). As genotoxic damage to the thyroid at the dosages applied is likely improbable, (as discussed below) such rodent thyroid tumors are conceived to result from continuous interference with the pituitary-thyroid hormonal homeostasis of rats and considered not likely relevant to humans.

In summary, in the described study in Wistar Han rats the observed tumor responses essentially were limited to the thyroid and mammary glands. Both of these target tissue-specific responses have rat-specific modes of action, not likely predictive for human cancer risk (Maronpot et al. 2015).

#### Evidence from epidemiology

A great number of epidemiological studies have been carried out throughout the years to investigate potential associations of exposure to AA with enhanced human cancer risk. According to EFSA (European Food Safety) (2015) the totality of epidemiological evidence from this wide range of human studies has not provided consistent indication for an association between AA exposure and increased cancer risk.

Few studies have utilized exposure biomarkers to approach more dependable dosimetry of AA. However, in most studies probable coexposure to other PRC has largely been disregarded. An unexpected recent discovery may have a greater bearing on the outcome of such epidemiological studies. Detailed monitoring of exposure biomarkers in carefully controlled human intervention studies has provided compelling evidence that certain PRC are continuously biosynthesized in the human body. For AA, this endogenous background exposure has been estimated to be relatively close to the average dietary exposure. Moreover, human endogenous exposure to the close AA analogue, acrolein (AC), has recently been reported to be even more than tenfold higher than that to endogenous AA (Goempel et al. 2017; Goerke et al. 2019; Ruenz et al. 2016, 2019). It may be presumed that such endogenous exposure may be variable, depending on as yet largely unknown factors of influence. The potential relevance of these findings, especially concerning the power to discover potential associations between AA exposure and human health risk, has yet to be addressed.

Continuous endogenous background exposure likely is not confined just to AA and AC but may comprise further electrophiles, arising from intermediary energy metabolism and/or from the human gut microbiome, including, for instance, several Michael reactive alkenals (Kiwamoto et al. 2015). Future epidemiological studies should take note of the consequences regarding the dosimetry of total human exposure. Thus, for a more appropriate estimate of potential human health consequences, well designed epidemiological and/or nutritional intervention studies will require elaborate and comprehensive dosimetry of the whole spectrum of PRCs in food and, in parallel, of their endogenously generated congeners, preferentially based on monitoring appropriate exposure biomarkers.

Finally, the association between occupational exposure to AA and cancer risk has been studied extensively, with longterm follow-up in 3 occupational cohorts of AA exposed workers (Collins et al. 1989; Marsh et al. 1999, 2007; Sobel et al. 1986; Swaen et al. 2007). These studies have shown no increased mortality from cancer overall or from specific cancer types, nor did they provide support for a positive dose–response among workers with respect to cumulative exposure and its duration or other exposure metrics (Pelucchi et al. 2011).

Overall, workers exposed at various working places to enhanced levels of AA have shown increased risk of mostly peripheral neurotoxicity, but no indication for an enhanced occupational cancer risk (summarized in EFSA 2015).

#### Genotoxicity and mutagenicity

The potential of an agent to damage DNA either directly by covalent binding or indirectly by otherwise altering its function and integrity commonly is referred to as genotoxic potential. The classical default position for regulatory authorities in cases of compelling evidence for genotoxicity was that there is no acceptable level of exposure (Gooderham et al. 2020). A more refined evaluation of the associated health risk however not only evaluates data on the genotoxic hazard, but also potential exposure, the dose–response relationship, and the key mode(s) of action, with emphasis on realistic consumers exposure scenarios.

#### *In*-*vitro* data

It is generally accepted that AA by itself is not a genotoxic and/or mutagenic agent at biologically relevant concentrations. To become active, it is supposed to require oxidative biotransformation into GA, primarily mediated by Cyp 450 2E1 (see Fig. 1) GA is assumed to act as the key genotoxic metabolite (Calleman et al. 1990; Segerbäck et al. 1995; Sumner et al.^1999^; Settels et al. 2008). GA has been shown to exert genotoxic and mutagenic effects in bacterial and mammalian test systems, whereas AA was found devoid of such activities at biologically relevant concentrations (Baum et al. 2005a, b; 2008; Hashimoto and Tanii 1985; Jiang et al. 2007; Koyama et al. 2006; Lamy et al. 2008; Mei et al. 2008; Puppel et al. 2005; Thielen et al. 2006).

GA, the activated genotoxic metabolite of AA, was tested in comparison with preactivated forms/model compounds of potent genotoxic and carcinogenic PRCs, using the comet assay in human blood/peripheral human lymphocytes as well as measuring induction of mutations and chromosomal damage.

In a first experiment, the potential of AA and GA to induce forward gene mutations in V79 Chinese hamster fibroblasts was compared using the hypoxanthine phosphoribosyl-transferase (*hPRT)* gene mutation assay in V79 cells. Cells were treated with AA (100–10 000 µM) or GA (400–2000 µM) for 24 h. AA did not induce mutations up to extremely, unrealistically high concentrations (6–10 mM). With GA, significantly elevated mutation frequencies became detectable only from 800 µM upwards. By contrast, the positive control N-methyl-N-nitro-N-nitroso-guanidine (MNNG), a potent and directly acting NOC, exerted within just 15 min of incubation marked mutagenic activity already at a concentration of 0.5 µM, orders of magnitude lower GA (Baum et al. 2005a).

In further experiments, a modified form of the comet assay was used, encompassing incubation of compounds in human whole blood (Baum et al. 2005a, b; Thielen et al. 2006). Whole blood may be considered as an ex vivo model system, containing a spectrum of proteins and other biomolecules with nucleophilic groups that may contribute to at least partially scavenging AA and GA by covalent binding after absorption from the gut. After 1 h incubation of human blood with 2,3,^14^ C-AA (30 µM), about 30% of the radiolabel was found in erythrocytes, 50% in protein-free plasma, and about 12–15% in plasma proteins (Bertow 2009). Thus, direct binding to noncritical blood constituents contributes to consuming a substantial portion of the bioavailable AA and its metabolite GA.

In this system the genotoxic/mutagenic activity of GA was compared with that of other activated/direct acting forms of known carcinogens: in addition to MNNG the benzo[a]pyrene metabolite (±)-anti-benzo[a]pyrene-7,8-dihydrodiol-9,10-epoxide (BPDE) and activated forms of N-Nitroso compounds-, including alfa-acetoxy-N-nitroso-diethanolamine (A-NDELA) and (3-N-nitroso-oxazolidine-2-one, NOZ-2. Induction of micronuclei (MN) was measured by incubating phytohemagglutinin treated blood for 23 h with AA (500–5 000 µM) or GA (50–1 000 µM) or with appropriate concentrations of the described activated/directly acting forms of carcinogens. In short, these studies confirmed the presumption of GA being a genotoxic and mutagenic agent of a rather low potency. About 300 µM of GA (4 h) were required to induce detectable DNA damage. By contrast, preactivated NOCs (3-N-nitroso-oxazolidine-2-one, NOZ-2 and A-NDELA) as well as a preactivated PAH, ((±)-anti-benzo[a]pyrene-7,8-dihydrodiol-9,10-epoxide (BPDE) exerted very much stronger genotoxic activity than GA, inducing significant DNA damage after short incubation times (15 min) at concentrations of 3 µM, orders of magnitude lower than GA. Likewise, in the *hPRT* mutagenicity test in V79 cells, GA-induced mutations only at concentrations of 800 µM and above, whereas preactivated NOCs and PAH significantly induced *hPRT* mutations already at more than 200 fold lower concentrations (Baum et al. 2005a, b; 2008; Thielen et al. 2006).

In a modification of the comet assay, lymphocyte DNA was additionally processed with the DNA repair enzyme formamido-pyrimidine-DNA-glycosylase (FPG) (Thielen et al. 2006). This can lead to an enhancement of strand breaks at positions where FPG recognizes apurinic and apyrimidinic sites, ring-opened pyrimidines (formamido-pyrimidines), and oxidized purines, representing DNA lesions conceived to result from oxidative DNA damage. They may, however, also arise as a consequence of DNA *N*^7^ Gua alkylation. Whereas in the comet assay without additional FPG treatment GA induced significant DNA damage in lymphocytes only at 300 µM and above, additional FPG processing led to the detection of DNA strand breaks already after 4 h incubation with 10 µM GA. This may reflect oxidative damage and/or transient formation of DNA *N*^7^-GA-Gua adducts which may subsequently generate apurinic sites (AP sites) via spontaneous or enzymatic depurination. AP sites are rapidly converted into strand breaks under the strongly alkaline comet assay standard conditions. Although therefore this individual experiment indicated some DNA interaction of GA at a lower concentration, GA induced DNA lesions are conceived to be rapidly repaired before eventually causing mutations. This presumption is compellingly supported by the above-reported findings. Direct DNA damage in lymphocytes became detectable by the comet assay only after incubation of blood with GA concentrations at or above 300 µM, in accordance with the *hPRT* mutagenicity test in V79 cells, becoming positive only at elevated concentrations of 800 µM and above. It may thus be inferred that at realistically low blood concentrations associated with dietary AA exposure, induction of DNA damage and mutations is not expected to take place. This presumption is further supported by taking into account that in vivo during the first pass through the liver, detoxifying GS coupling kinetically outperforms CYP mediated oxidative GA formation, as demonstrated in primary rat hepatocytes (Watzek et al. 2013).

At this point, it may be allowed to comment on certain quality criteria required to evaluate the merits of in vitro or in vivo studies and model experiments aimed to contribute to human risk assessment. As a major element of quality, exposure conditions chosen for such experiments need to be justified with respect to animal and human exposure, and toxicokinetics. *In*-*vitro* studies utilizing unrealistically high concentrations (i.e. any concentration markedly exceeding in vivo blood/tissue levels associated with worst-case environmental exposure) to become meaningful require compelling proof that relevant MOAs and toxicokinetics are not altered. For example, studying mutational signatures in human embryonal cells by 24 h treatment at mM concentrations of AA/GA for 24 h appears far away from physiological conditions. An interpretation of the results obtained under such extreme conditions as revealing “widespread contribution of acrylamide to carcinogenesis in humans “therefore is not convincing (Zhivagui 2019)”.

#### Taken together with the totality of the above described in vitro evidence compellingly reveals

1. AA not to be genotoxic/mutagenic and
2. GA to exert at best very weak genotoxicity and mutagenicity (especially under biologically relevant conditions), compared to preactivated forms of established food borne mutagens and carcinogens.

The conclusion that GA is to be considered as a very low potency genotoxic and mutagenic agent can also be reconciled with its preferential DNA *N*^7^-Gua alkylation. The propensity of DNA lesions to lead to mutations is dependent on several parameters, such as the type and topology of the lesion, the rate and correctness of DNA repair processes, induction of apoptosis, and proliferative response. As an overall consequence, the mutagenic potential of different DNA lesions ranges over several orders of magnitude (Nestmann et al. 1996). DNA *N*^7^-Gua alkyl adducts are known to be frequently formed but may have minimal biological relevance since they are chemically unstable and do not participate in Watson–Crick base pairing. Thus little to no evidence has been ascribed to *N*^*7*^-Gua alkyl adducts, as a noteworthy cause of mutations in cells and tissues (Boysen et al. 2009).

#### *In*-*vivo* studies

The following discussion concentrates on studies at realistic dietary AA exposure levels, i.e. those that may be expected from the intake of heat-processed foods. Data from such experimental studies in animals have been complemented by carefully controlled human intervention studies investigating the effects of dietary AA on human biomarker kinetics (Goempel et al. 2017; Goerke et al. 2019; Ruenz et al. 2016).

Early on after the discovery of AA as a PRC in food, the question of its bioavailability from the food matrix during digestion became relevant. Thus, in a systematic study in Sprague–Dawley (SD) rats, the bioavailability of AA from different food matrices was tested in comparison to AA from ingested drinking water.

AA was given to rats by feeding foods containing known amounts of AA (generated by heat processing) for up to 9 days, resulting in a dietary exposure of 50 or 100 μg AA/kg bw/day (Berger et al. 2011). Positive controls received the same dosages of AA in drinking water, negative controls just water. Short term and long-term exposure biomarkers were monitored, including urinary MAs and Hb adducts of AA and GA to the N-terminal valine of Hb. Plasma levels of AA and GA were monitored as well and induction of DNA damage in white blood cells and in liver cells (hepatocytes) was investigated using the in vivo comet assay.

Significant differences in overall bioavailability in terms of the area under the curve (AUC) values of AA ingested from water and different food matrices were not observed. Plasma kinetics of AA essentially showed peak values 30 min after intake, yet with about fourfold higher peak levels in animals after AA intake in water as compared to foods. The delayed plasma kinetics observed after food intake, reflecting delayed liberation from the various food matrices during digestion, lead however to about the same terminal AUC levels as from water, resulting in similar overall bioavailability (Berger et al. 2011).

Formation of Hb adducts of AA depicted linear time- and exposure-related dose–response, showing treatment associated cumulative buildup of AA Hb adducts in blood erythrocytes. In contrast, Hb adducts of GA were not found significantly enhanced above untreated control. This was remarkable since the urinary short time exposure biomarker of metabolically formed GA, the mercapturic acid *N*-acetyl-S-(2-carbamoyl-2-hydroxyethyl)cysteine (GAMA) indicated significant GA generation in the liver.

This result suggested that at the AA dose levels ingested, any GA formed metabolically in the liver was effectively scavenged by glutathione coupling (Berger et al. 2011).

Of note, the genotoxic AA metabolite GA was only detected at one single sampling point (4 h) in plasma in minute concentration, close to the detection limit (0.06 µM). This supports the interpretation that at dietary exposure level, GA, as soon as it is metabolically formed in the liver, is directly and practically quantitatively scavenged by GSH coupling. The GSH adduct is metabolically further processed into the corresponding mercapturic acid and excreted in the urine, thus allowing biomarker-based dosimetry of the GA formed from AA at first pass in the liver. As a further confirmation of absent genotoxicity, the comet assay values did not show any enhanced DNA damage in liver or blood cells under any dietary exposure condition, compared to untreated control (Berger et al. 2011).

In a subsequent dose–response study AA was given orally in single doses of 0.1–10 000 μg/kg bw to female SD rats (Watzek et al. 2012a). The lowest dose (0.1 µg/kg bw) was below average dietary consumers’ exposure (0.4–0.6 µg/kg bw) the next higher dose (1 µg/kg) close to this level. Further dose escalation was achieved by factors of 10, up to the highest dose. Formation of urinary MAs and of *N*^7^-GA-Gua DNA adducts in liver, kidney, and lung was measured 16 h after application, a time point where a previous pilot experiment had shown maximal.

#### *N*^7^-GA-Gua DNA concentration to occur

Although mercapturic acid monitoring clearly reflected escalating dose-related responses, DNA-*N*^7^-GA-Gua adduct formation behaved quite differently. Enhanced formation in comparison to untreated control was not detectable in any organ tested at 0.1 μg AA/kg bw. At a dose of 1 μg/kg bw, adducts were found in the kidney (about 1 adduct/10^8^ nucleotides) and lung (below 1 adduct/10^8^ nucleotides), but not in liver. At 10 and 100 μg/kg bw, respectively, adducts were found in all three organs, however at levels close to those found at 1 μg AA/kg, in a range of about 1–2 adducts/10^8^ nucleotides. Thus, in the dose range from 0.1 to 100 µg/kg bw/d no linear dose response relationship was apparent in any organ tested, although the doses were escalated by a constant factor of 10.

Of note, earlier results from the administration of higher single oral doses of AA to rodents have shown a relatively even distribution of DNA adducts in a spectrum of organs. *N*^7^-GA-Gua adduct concentrations found in liver DNA of AA treated mice have been shown to be comparable to those determined in tumor target tissues. Altogether, this does not support the premise that induction of DNA damage is relevant for the neoplastic effects of AA in target organs of experimental animals (Doerge et al. 2005; Gamboa da Costa et al. 2003; Ghanayem et al. 2005; Manière et al. 2005).

Remarkably, MA excretion in urine of untreated controls indicated some background AA exposure of presumed endogenous origin in SD rats. This was concluded from the quantitative comparison of AA intake in food with the urinary mercapturic acid output. Control rats were estimated to ingest with their experimental diet at best 0.4 nmol/day (0.1 µg/kg bw). This estimate was based on a maximum daily uptake of 30–50 g of the experimental diet with an AA content of 0.5 µg/kg (in fact it was below because 0.5 µg/kg was the limit of detection). The total MA urinary output reached about 0.8 nmol/day, estimated about equivalent to 0.6–0.7 µg/kg bw of AA exposure, thus substantially exceeding the maximum supposed AA intake (Watzek et al. 2012a).

The endogenous background found in SD rats is of similar magnitude as has been reported subsequently for humans where sustained endogenous background exposure was discovered and was estimated to be relatively close to the average dietary consumers’ exposure (Goempel et al. 2017; Goerke et al. 2019; Ruenz et al. 2016; Watzek et al. 2012b).

It appears meaningful at this point to put the above-reported DNA adduct yields (1–2 adducts in 100 million nucleotides) into perspective, by reference to the human background levels reported for various tissues or cells. DNA adduct levels reflecting background exposure to genotoxic agents of various origin have been found in human tissues at levels of up to about 200 adducts in 100 million (10^8^) nucleotides (Nakamura et al. 2014; Swenberg et al. 2011). Considering adducts structurally more closely related to *N*^7^-GA-Gua, such as *N*^7^-2-carboxyethyl-Gua, *N*^7^-2-hydroxyethyl- or *N*^7^-methyl-Gua, aggregated background levels may together make up for a range of about 50–100/10^8^ nucleotides (summarized in Watzek et al. 2012a; Hartwig et al. 2020).

It thus may be inferred that up to the dose of 100 µg/kg bw which substantially (by a factor at least 100) exceeded human average dietary exposure levels, there was no indication for a dose-related induction of DNA damage in rats. GA specific DNA adduct levels remained at the lower bound of closely related human background DNA adducts.

#### Evidence from toxicogenomic and transcriptional profiling data

Several toxicogenomic studies in rodents exposed up to relatively high doses of AA were conducted and gene expression profiling was performed to approach elucidation of key MOA(s) (Chepelev et al. 2017, 2018; Recio et al. 2017). They are of interest because they address potential MOAs in the main rodent target tissues of AA-induced neoplastic changes, the thyroid, the testes, and the Harderian gland.

Taken together, the results of these transcriptional profiling studies did not show alterations of gene expression profiles indicative for pathways associated with putative genotoxic MOAs such as those involving p53 homeostasis, DNA repair, or the cell cycle orchestrating network. Instead, the findings pointed to alternative MOA(s). In animals exposed to dosages of 1.5–24.0 mg/kg bw AA in drinking water for up to 31 days, pronounced effects on genes involved in calcium signalling and cellular transport were observed, concomitant with cytoskeletal processes in target tissues. However, no evidence supporting a genotoxic MOA became apparent, even at the rather high dosages applied (Chepelev et al. 2017, 2018; Recio et al. 2017).

Overall, therefore, perturbation of calcium signalling pathways was discovered as a potential key event for AA-mediated neoplastic transformation in these rodent tissues. The biomolecular details of how AA interferes with cellular calcium signalling and homeostasis to finally bring about neoplastic transformation are not clear yet.

In support of the premise that genotoxicity is not a probable MOA of AA, a further study in F 344 rats given AA at dosages up to 12.0 mg/kg bw/day for various subchronic time periods has shown no significant increase in either mutation of the *Pig*-a (phosphatidylinositol glycan, class A) gene, nor induction of micronuclei. Whereas at exceedingly high dosage levels of up to 24.0 mg/kg bw/day in mice some increase in micronuclei was observed, no *Pig*-a gene mutations were recorded (Hobbs et al. 2016). In addition, at dosages < 6.0 mg/kg/day no in vivo mutagenicity was observed, in agreement with the perception of a non-genotoxic MOA for AA-induced tumorigenicity in rodents (Hobbs et al. 2016).

Taken together, these results are in line with those mentioned above and not supportive for a key role of genotoxicity in the mode of action of AA.

## Conclusions

The totality of present-day evidence does not support the premise that AA induces malignant transformation in animal experiments by virtue of a genotoxic MOA. Whereas AA itself undoubtedly is nongenotoxic, it can be converted metabolically to the epoxide GA which may exert DNA damage by covalent binding. It has been presumed that such genetic damage may result in fixed mutations, eventually leading to neoplastic transformation. Although this view has been favoured in the past as the most probable key event underlying AA-induced neoplastic transformation, compelling evidence is lacking. As summarized here, the genotoxicity of AA may rather be understood as an effect occurring, if at all, at exceedingly high dose levels, not relevant to realistic physiological conditions, especially not to those prevailing at consumers’ dietary exposure level. Thus, whereas genotoxicity does not appear to be a key MOA relevant for neoplastic transformation by AA, there is scientific evidence for a non-genotoxic MOA to be responsible for tumor formation in rodents. Tumors found enhanced in rodents are, however, considered to be strain and species-specific, not likely predictive for humans.

Taken together, this may argue for the establishment of an alternative, MOA based point of departure (POD) for risk assessment and/or the definition of a tolerable daily intake level (TDI) on the basis of a compellingly established no observed adverse effect (NOAEL) level.

Summarizing the major arguments, it is concluded that

- The presumed genotoxic key metabolite of AA, GA is a rather poor mutagen, predominantly inducing *N*^7^-GA-Gua lesions, known to be of rather low (or even absent) mutagenic activity at biologically relevant doses.
- In-vivo, at realistic low exposure levels encompassing diet-related intake, AA induces only very minor DNA damage in rats. At single dosages up to at least 100 μg/kg bw (which strongly exceeds present-day average consumer exposure), DNA damage was not found dose-related and remained at the lower bound of human background DNA damage of comparable DNA *N*^7^-Gua lesions.
- Repeated intake of AA in foods/water at dosages of 50–100 µg/kg bw resulted in linear time- and exposure-related cumulative buildup of AA Hb adducts in blood erythrocytes. However, Hb-GA adducts were not found enhanced, although the mercapturic acid GAMA indicated significant GA formation in the liver. This allows us to conclude that up to the AA dose levels ingested, any GA formed metabolically in the liver will be scavenged by glutathione coupling. This conclusion is further supported by in vitro data in primary hepatocytes demonstrating the detoxifying formation of GSH adducts to be up to about 3 times faster than GA formation.
- Recent evidence from toxicogenomic studies argues for MOAs other than genotoxicity. This applies especially to the target organs of AA in rodent carcinogenicity studies, such as thyroid, testes, and Harderian gland, where pronounced effects on calcium signalling and on cytoskeletal functions have been observed, however, no compelling evidence was found to support a genotoxic MOA.
- Longterm studies on neoplastic effects of AA have revealed the enhanced formation of mammary gland adenomas and fibroadenomas, thyroid follicular cell neoplasms, and testicular mesotheliomas in F344 rats and in mice tumors in the Harderian gland, mammary gland, lung, ovary, skin, and stomach/forestomach. At variance, in another rat strain (Wistar Han rats) tumor responses were largely limited to the thyroid and mammary glands. These rodent neoplasms may be considered as species- and strain-specific responses with specific modes of action not likely predictive for human cancer risk.

As an overall conclusion, the totality of presently available scientific evidence clearly argues against a genotoxic MOA underlying the neoplastic effects of AA, considered more likely to arise as sequelae of toxic effects, as exemplified by interference with the cellular calcium signalling system in target tissues.
